# miR-708-3p targetedly regulates LSD1 to promote osteoblast differentiation of hPDLSCs in periodontitis

**DOI:** 10.1007/s10266-024-00963-9

**Published:** 2024-07-03

**Authors:** Qing Shao, ShiWei Liu, Chen Zou, YiLong Ai

**Affiliations:** 1 Department of Orthodontics, Foshan Stomatological Hospital, School of Stomatology and Medicine, Foshan University, No.5 Hebin Road, Chancheng District, Foshan, 528000 Guangdong China; 2 Department of Stomatology, Foshan First People’s Hospital, Foshan, 528000 Guangdong China

**Keywords:** miR-708-3p, LSD1, Periodontitis, Inflammation, Osteogenesis

## Abstract

Periodontitis (PD) is a multifactorial inflammatory disease associated with periodontopathic bacteria. Lysine-specific demethylase 1 (LSD1), a type of histone demethylase, has been implicated in the modulation of the inflammatory response process in oral diseases by binding to miRNA targets. This study investigates the molecular mechanisms by which miRNA binds to LSD1 and its subsequent effect on osteogenic differentiation. First, human periodontal ligament stem cells (hPDLSCs) were isolated, cultured, and characterized. These cells were then subjected to lipopolysaccharide (LPS) treatment to induce inflammation, after which osteogenic differentiation was initiated. qPCR and western blot were employed to monitor changes in LSD1 expression. Subsequently, LSD1 was silenced in hPDLSCs to evaluate its impact on osteogenic differentiation. Through bioinformatics and dual luciferase reporter assay, miR-708-3p was predicted and confirmed as a target miRNA of LSD1. Subsequently, miR-708-3p expression was assessed, and its role in hPDLSCs in PD was evaluated through overexpression. Using chromatin immunoprecipitation (ChIP) and western blot assay, we explored the potential regulation of osterix (OSX) transcription by miR-708-3p and LSD1 via di-methylated H3K4 (H3K4me2). Finally, we investigated the role of OSX in hPDLSCs. Following LPS treatment of hPDLSCs, the expression of LSD1 increased, but this trend was reversed upon the induction of osteogenic differentiation. Silencing LSD1 strengthened the osteogenic differentiation of hPDLSCs. miR-708-3p was found to directly bind to and negatively regulate LSD1, leading to the repression of OSX transcription through demethylation of H3K4me2. Moreover, overexpression of miR-708-3p was found to promote hPDLSCs osteogenic differentiation in inflammatory microenvironment. However, the protective effect was partially attenuated by reduced expression of OSX. Our findings indicate that miR-708-3p targetedly regulates LSD1 to enhance OSX transcription via H3K4me2 methylation, ultimately promoting hPDLSCs osteogenic differentiation.

## Introduction

Periodontitis (PD) is a persistent inflammatory ailment that results in the degradation of periodontal tissue, ultimately culminating in tooth loss [1, 2]. It is characterized by irreversible damage to periodontal hard and soft tissues, and periodontal homeostasis [3]. The pathologic processes of PD are the result of interactions between periodontal bacteria and host immune responses [4, 5]. Current management options for PD involve expensive therapies such as mechanical debridement, flap curettage, scaling, and root planning [6, 7]. However, these treatments can only prevent the development of PD and cannot regenerate lost periodontal tissues [8]. Therefore, identifying a biological target could be beneficial for the amelioration of PD.

Derived from the periodontal ligament, human periodontal ligament stem cells (hPDLSCs) are a type of mesenchymal stromal cells (MSCs) with the unique ability to differentiate into osteoblasts in vitro [9]. This unique feature positions hPDLSCs as a prime candidate for treating PD [10]. Histone demethylase significantly affects periodontal genes and inflammation, growth, and treatment of periodontal tissues [11]. Lysine-specific demethylase 1 (LSD1), a flavonoid-dependent demethylase, involves in regulating gene expression, immune response, and inflammatory symptoms in oral diseases [12]. In addition, as a key regulator of osteogenesis, it participates in bone construction programming by interacting with miRNAs [13].

miRNA is a short, non-coding RNA that modulates gene expression in eukaryotic cells [14]. The dynamic equilibrium present within the skeletal system is vital for maintaining healthy bone tissue, but this balance can be disrupted by miRNAs [15]. Abnormal expression of miRNAs during bone metabolism has been strongly linked to various bone diseases [16]. Upon induction of osteogenesis, miR-708-3p was upregulated to modulate bone metabolism and alleviate bone diseases [17]. However, the relationship between miR-708-3p and osteogenic differentiation of hPDLSCs remains unclear.

Researchers recently discovered the correlation between trimethylation of di-methylated H3K4 (H3K4me2) and gene activation in MSCs, specifically in relation to osteogenic differentiation [18, 19]. LSD1, which is enriched in the osterix (OSX) promoter, can cause H3K4me2 demethylation and downregulation of OSX [20]. OSX serves as a pivotal transcription factor imperative for the differentiation of osteoblasts. A preceding investigation demonstrated that augmenting OSX not only fosters the growth of human dental pulp cells but also effectively mitigates inflammation in pulpitis [21]. Considering the aforementioned considerations, our objective is to delve into the influence of miR-708-3p on the progression of PD, specifically by orchestrating the recruitment of LSD1 into the OSX promoter region, thereby epigenetically suppressing OSX expression. To achieve this goal, we established an in vitro periodontitis model of hPDLSCs through LPS induction. Subsequently, we conducted molecular biology experiments to validate the regulatory roles of miR-708-3p and LSD1 on the osteogenic differentiation of hPDLSCs within an inflammatory environment. Lastly, we delved into the potential molecular mechanisms underlying the involvement of miR-708-3p in the progression of PD from epigenetic perspective.

## Materials and methods

### hPDLSCs extraction and identification

The research was carried out following the principles of the Declaration of Helsinki and was approved by the Ethics Committee of Foshan Stomatological Hospital protocol. Signed informed consent was also obtained from all participants or guardians. Premolars extracted for orthodontic purposes between the ages of 14–18 years without dental caries or periodontal disease were used for the study. Periodontal ligament tissue was extracted from the middle of the tooth root, washed with PBS, and then subjected to digestion with 3 mg/mL collagenase I and 3 mg/mL dispose II at 37 °C for 1 h. The resulting tissue was evenly distributed in a 6 cm culture dish and maintained in a CO_2_ incubator at 37 °C with α-MEM medium containing 10% FBS (Gibco, USA), which was refreshed every 3 days. Following filtration through a 70 µm cell strainer, a single cell suspension was obtained, and the cells were detached with 3 mg/mL trypsin (Gibco, USA) and passaged at a 1:2 ratio when they reached 80% confluency. The third passage cells were used for subsequent experiments. Stem cell characteristics were evaluated using flow cytometry with FITC-labeled CD34, CD45, and CD90 (all from Abcam, USA).

### hPDLSCs treatment and transfection

To mimic the microenvironment of PD, hPDLSCs were treated with 10 µg/ml *Porphyromonas gingivalis* LPS (PG-LPS, Sigma, USA) as previously reported [22]. The small interfere (si)-LSD1, miR-708-3p mimic, si-OSX and its corresponding control were obtained from Guangdong Ruibo Biotechnology Co., LTD. The hPDLSCs were cultured in osteogenic induction medium (OIM) that was replaced every 3 days.

### Alizarin red staining

Following a 21-day incubation period, the cells on the plates were fixed with 2 mL of 4% paraformaldehyde per well and stained with alizarin red solution. After 30 min, the plates underwent five washes with PBS to eliminate any surplus dye. The cells were then examined, scanned, and photographed under a microscope. To determine the amount of mineralized matrix deposition, the mineral nodules were dissolved using 10% cetylpyridinium chloride (Solarbio, China), and the OD562 value was measured using a microplate reader.

### ELISA

After different treatments, we gathered cell supernatants from hPDLSCs. Subsequently, the concentrations of TNF-α, IL-6, and IL-1β in these cell supernatants were determined through ELISA assays following the guidelines provided by the ELISA kit (Beyotime Biotechnology, China).

### Dual-luciferase reporter gene assay

LSD1-WT and LSD1-MUT reporter vectors were generated by incorporating the binding sites of WT and MUT miR-708-3p with LSD1 fragments in the pmirGLO vector. These vectors were then transfected into 293 T cells using Lipofectamine 2000 (Qiagen, USA). 48 h later, cells were collected using a passive lysis buffer. Luciferase activity was subsequently measured using a GloMax® 20/20 luminometer (Promega, USA).

#### Cytoplasmic and nuclear miR-708-3p analysis

Cytoplasmic and nuclear fractionation were performed using PARIS™ Kit (Thermo Fisher, USA) following the instructions provided by the manufacturer. Briefly, hPDLSCs were collected and resuspended in pre-chilled cell fractionation buffer (200 μL), followed by centrifugation at 500 rpm for 5 min. Subsequently, RNAs were isolated, and the expression of miR-708-3p was assessed using qPCR, with U6 and GAPDH serving as reference genes.

### Chromatin immunoprecipitation (ChIP) assay

The experimental procedure was performed using the chromatin immunoprecipitation kit (Millipore, USA). hPDLSCs were transfected with si-LSD1 or si-NC and subsequently cultured in OIM for 14 days. The cells were crosslinked with 1% formaldehyde for 10 min at 37 °C and then suspended in a cell lysis buffer (50 mM Tris–HCl [pH 8.1], 10 mM EDTA, 1% SDS). Following processing, the cells were lysed using a cell lysis solution, and the resulting samples were diluted tenfold with an immunoprecipitation dilution buffer. Immunoprecipitation was performed at 4 °C, and the lysate was incubated overnight with anti-LSD1 antibody (Abcam, USA), anti-H3K4me2 antibody (Abcam, USA), or nonspecific rabbit IgG. The immunocomplex was incubated with Sepharose CL-4B at 4 °C for 2 h. After multiple washes, the DNA fragments were eluted and purified, and the DNA precipitate was subjected to PCR amplification.

### Quantitative PCR (qPCR)

Total RNA was extracted using TRizol (Invitrogen, USA) and reverse-transcribed into cDNA using Tiangen Biotechnology (China) kit. PCR was conducted with small RNA U6 and GADPH as internal control, using 2 × SYBR Green QPCR Master Mix from Shanghai Dongsheng Biotechnology (China). The relative gene expression was determined using the 2^−ΔΔCt^ method. All primer sequences are provided in Table 1.

**Table 1:** Primer sequence

Gene	GenBank accession	Forward (5’ → 3’)	Reverse (5’ → 3’)	Product length (bp)
LSD1	NM_015013	CCTGAAGAACCATCGGGTGT	CCTTCTGGGTCTGTTGTGGT	124
miR-708-3p	LM380490.1	AAAGAGACCGGUUCACUGUGA	UUUCUCUGGCCAAGUGACACU	132
U6	NM_14486	CTCGCTTCGGCAGCACA	AACGCTTCACGAATTTGCGT	91
GADPH	NM_001256799.1	CTGCACCACCAACTGCTTAG	TGATGGCATGGACTGTGG	94

### Western blotting

Protein extraction was performed using RIPA and PMSF from Shanghai Life Mode Engineering (China), and the protein concentration was determined using the BCA kit from Shanghai Dongsheng Biotechnology (China). The PVDF membrane (0.22 µm, Millipore ISEQ00010, USA) was then incubated with primary antibodies (listed in Table 2) overnight at 4 °C, followed by incubation with HRP-conjugated secondary antibodies (Abcam, USA). Protein bands were detected using Prime Western Blotting Detection Reagent (Cytiva, UK). A ChemiDoc MP imaging system (Tanon 4800, China) was used to detect chemiluminescence. Image J software was used to analyze the gray value of the bands.

**Table 2:** All antibodies in this study

Gene	Brand	Provenance	Article number
LSD1	Bioss	rabbit	bs-3821R
OSX	Abcam	rabbit	ab209484
Runx2	Bioss	rabbit	bs-1134R
OCN	Affinity	rabbit	DF12303
H3K4me2	Abcam	rabbit	ab32356
GAPDH	proteintech	mouse	60,004-1-Ig

### Statistical analysis

Experimental data were analyzed using GraphPad Prism 9.0 software and statistical tests were chosen based on the distribution and variance homogeneity of the data. For normally distributed and homogeneous variance data, *t *tests were used to compare two groups. For multiple group comparison, either LSD analysis of variance or Dunnett’s T3 test was used based on the distribution and variance. Measurement data were presented as mean ± standard deviation, and statistical significance was determined at* P* < 0.05.

## Results

### Inflammatory microenvironment led to increased expression of LSD1 in hPDLSCs

**Fig. 1:**
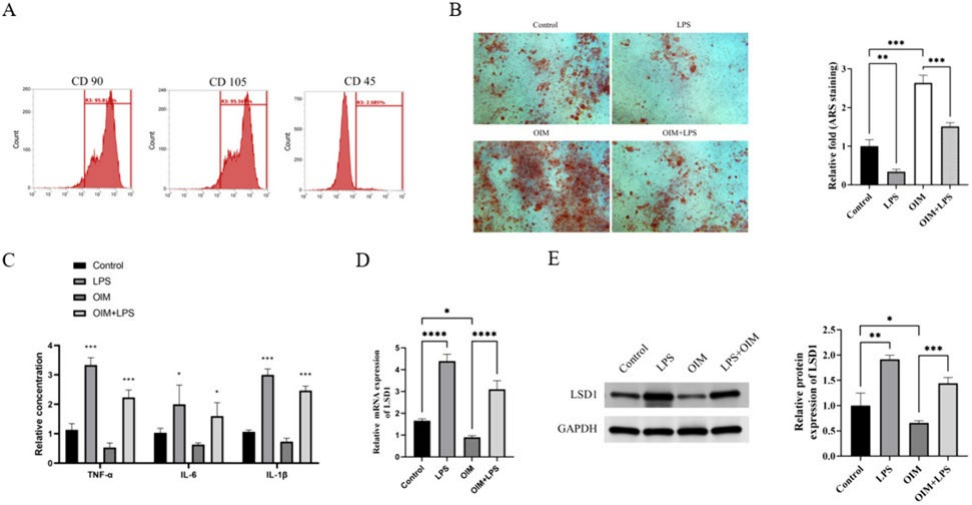
Inflammatory microenvironment increased LSD1 expression in hPDLSCs. **A** The levels of CD90, CD105, and D45 in hPDLSCs surface. **B** The mineralization levels of hPDLSCs in each group. **C** The content of TNF-α, IL-6, and IL-1β in cell supernatant in each group. **D** The mRNA levels of LSD1 in hPDLSCs in each group. **E** The protein levels of LSD1 in hPDLSCs in each group. The samples were from the same experiment, and the gel/imprint was processed in parallel. All stripes were cropped. *P < 0.05; ****P < 0.0001

Previous studies have established that LSD1 could promote osteogenic differentiation of stem cells [23]. However, the impact of LSD1 on hPDLSCs differentiation in PD remains unclear. We cultured and identified hPDLSCs using flow cytometry and found that they expressed CD105 and CD90, but not CD45 (Fig. 1A). We next cultured hPDLSCs in OIM, which resulted in a notable increase in mineralization levels. To mimic an inflammatory microenvironment, we exposed the hPDLSCs to LPS, and interestingly, we observed a reduction in OIM-induced mineralization in the presence of LPS (Fig. 1B). In our subsequent analysis, we quantified the levels of inflammatory factors in the supernatants of hPDLSCs. The findings revealed a noteworthy escalation in the release of TNF‐α, IL-6, and IL-1β following exposure to LPS (Fig. 1C). Furthermore, we observed that LSD1 expression increased with LPS treatment, but decreased with osteogenic induction. Furthermore, the LSD1 expression was found to be higher in LPS + OIM group compared to OIM group (Fig. 1D–1E). Overall, LSD1 expression elevated in inflammatory microenvironment, but decreased following osteogenic induction.

### The effects of LSD1 on hPDLSCs osteogenic differentiation in microenvironment of PD

**Fig. 2:**
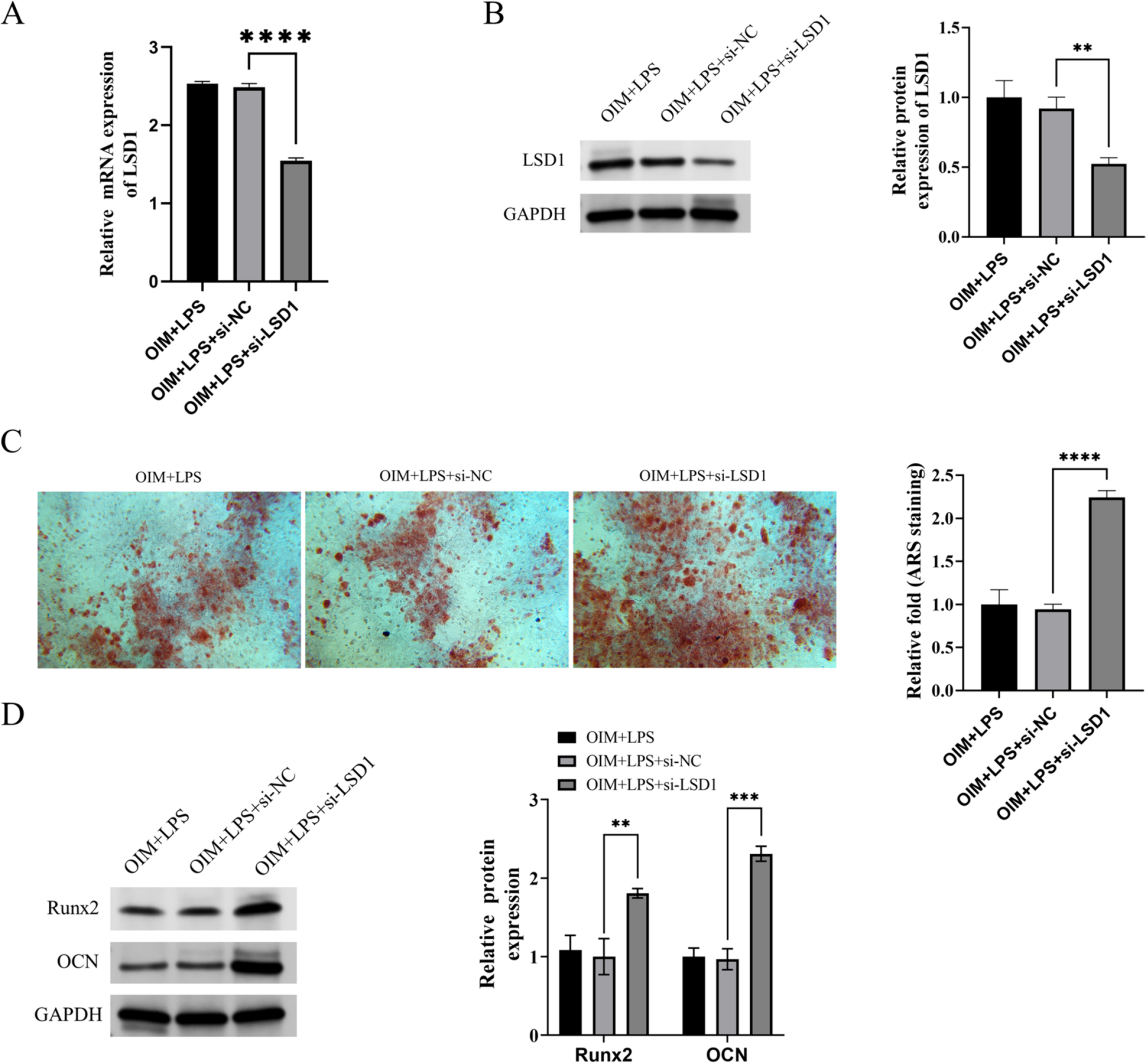
Inhibition of LSD1 impeded osteoblast differentiation in microenvironment of PD. **A** The mRNA levels of LSD1 in hPDLSCs in each group. **B** The protein levels of LSD1 in hPDLSCs in each group. **C** The mineralization levels of hPDLSCs in each group. **D** The protein levels of Runx2 and OCN in hPDLSCs in each group. The samples were from the same experiment, and the gel/imprint was processed in parallel. All stripes were cropped. ****P < 0.0001

We conducted a study where we transduced hPDLSCs with control siRNA or LSD1 siRNA to investigate the impact of LSD1 on hPDLSCs osteogenic differentiation in PD microenvironment. As a result of transfection, the expression of LSD1 was significantly reduced in hPDLSCs (Figs. 2A-2B). We subsequently performed alizarin red staining to assess mineralization levels, and the results showed that silencing LSD1 promoted hPDLSCs mineralization (Figs. 2C). Previous research has indicated that Runt-related transcription factor 2 (Runx2) and osteocalcin (OCN) serve as markers of osteogenic differentiation [24]. Our western blot results revealed that inhibition of LSD1 increased Runx2 and OCN protein expression in hPDLSCs (Figs. 2D).

### miR-708-3p bound to LSD1 to promote osteoblast differentiation of hPDLSCs in microenvironment of PD

**Fig. 3:**
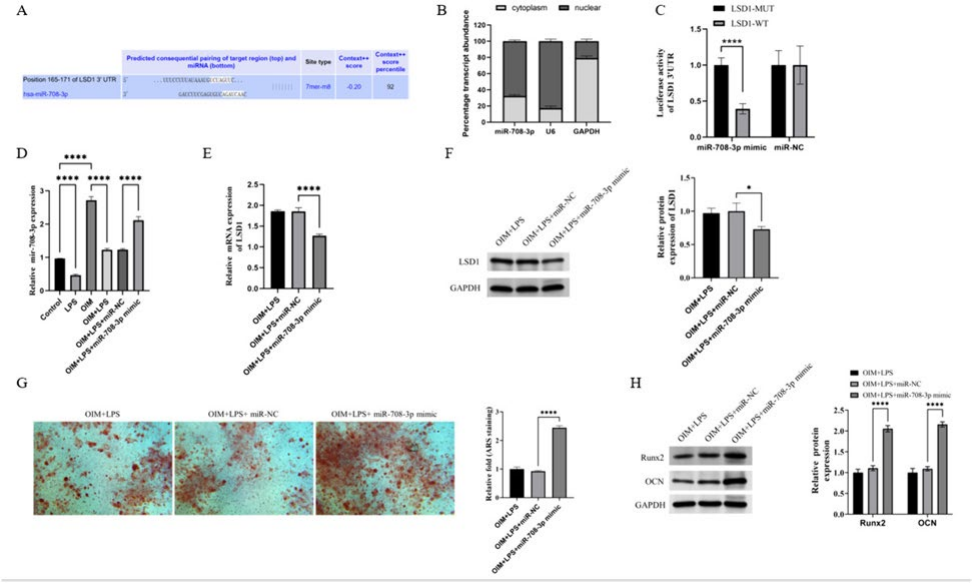
LSD1 promoted osteoblast differentiation of hPDLSCs by binding miR-708-3p in microenvironment of PD. **A** The binding sites between LSD1 and miR-708-3p. **B** The subcellular localization for miR-708-3p in hPDLSCs. **C** Fluorescence intensity of LSD1 3'UTR in hPDLSCs in each group. **D** The expression of miR-708-3p in hPDLSCs in each group. **E** The mRNA levels of LSD1 in hPDLSCs in each group. **F** The protein levels of LSD1 in hPDLSCs in each group. **G** The mineralization levels of hPDLSCs in each group. **H** The protein levels of Runx2 and OCN in hPDLSCs in each group. The samples were from the same experiment, and the gel/imprint was processed in parallel. All stripes were cropped. ****P < 0.0001

To explore upstream regulatory mechanism, we utilized TargetScan database to predict potential miRNAs that may be associated with LSD1 and identified miR-708-3p as a promising candidate miRNA (Figs. 3A). To investigate the potential interaction between miR-708-3p and LSD1, we conducted an analysis of the subcellular localization of miR-708-3p in hPDLSCs. The findings indicated that miR-708-3p is expressed in both the cytoplasm and the nucleus, with a significantly higher expression level observed in the nucleus (Fig. 3B). Subsequently, we validated that miR-708-3p binds to LSD1 (Fig. 3C). In addition, we observed that miR-708-3p was decreased following LPS treatment and increased following osteogenic induction (Figs. 3D). We subsequently introduced the miR-708-3p mimic to increase its expression. After transfection, we observed that miR-708-3p overexpression decreased LSD1 expression in hPDLSCs (Figs. 3E-3F). In addition, miR-708-3p overexpression enhanced the mineralization of hPDLSCs (Fig. 3G). Lastly, we found that miR-708-3p increased Runx2 and OCN protein expression (Fig. 3H).

### miR-708-3p targets LSD1 to increase OSX transcription through H3K4me2 demethylation

**Fig. 4:**
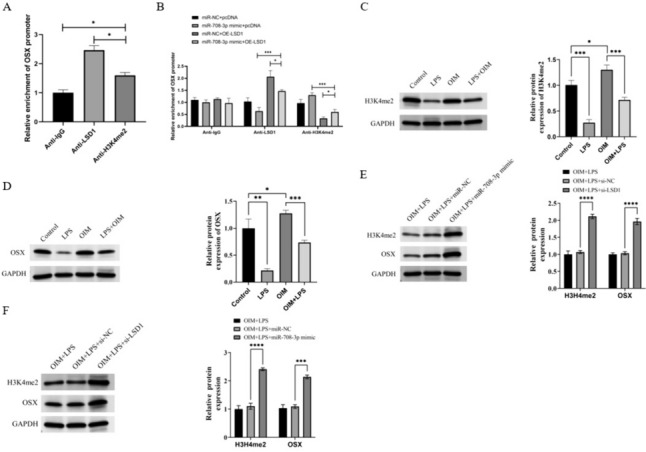
miR-708-3p-targeted LSD1 increased OSX transcription through H3K4me2 demethylation. **A** The enrichment of LSD1 and H3K4me2 in the promoter of OSX. **B** The enrichment of LSD1 and H3K4me2 in the promoter of OSX in each group. **C** The protein levels of H3K4me2 in hPDLSCs in each group. **D** The protein levels of OSX in hPDLSCs in each group. **E**–**F** The protein levels of H3K4me2 and OSX in hPDLSCs in each group. The samples were from the same experiment, and the gel/imprint was processed in parallel. All stripes were cropped. ****P < 0.0001

LSD1 specifically promotes H3K4me2 demethylation to regulate OSX transcription [24]. Therefore, we hypothesized that LSD1 represses OSX by promoting the demethylation of H3K4me2 in PD. Our ChIP assays revealed that LSD1 and H3K4me2 could bind to the OSX promoter, respectively (Fig. 4A). In addition, we observed a diminished OSX promoter enrichment in anti-LSD1 immunoprecipitations but an augmented enrichment in anti-H3K4me2 immunoprecipitations in the miR-708-3p mimic + OE-LSD1 group compared to the miR-NC + OE-LSD1 group (Fig. 4B). Next, we found that the expression of H3K4me2 was decreased after LPS treatment, but enhanced after osteogenic induction (Fig. 4C). The expression of OSX followed the same trend as H3K4me2 expression (Fig. 4D). Lastly, western blot results also confirmed that H3K4me2 and OSX expression were increased following LSD1 inhibition and miR-708-3p overexpression (Fig. 4E-4F). These findings suggest that miR-708-3p increased OSX transcription through LSD1-mediated H3K4me2 demethylation.

### miR-708-3p promoted osteoblast differentiation of hPDLSCs by increasing OSX transcription in microenvironment of PD

**Fig. 5:**
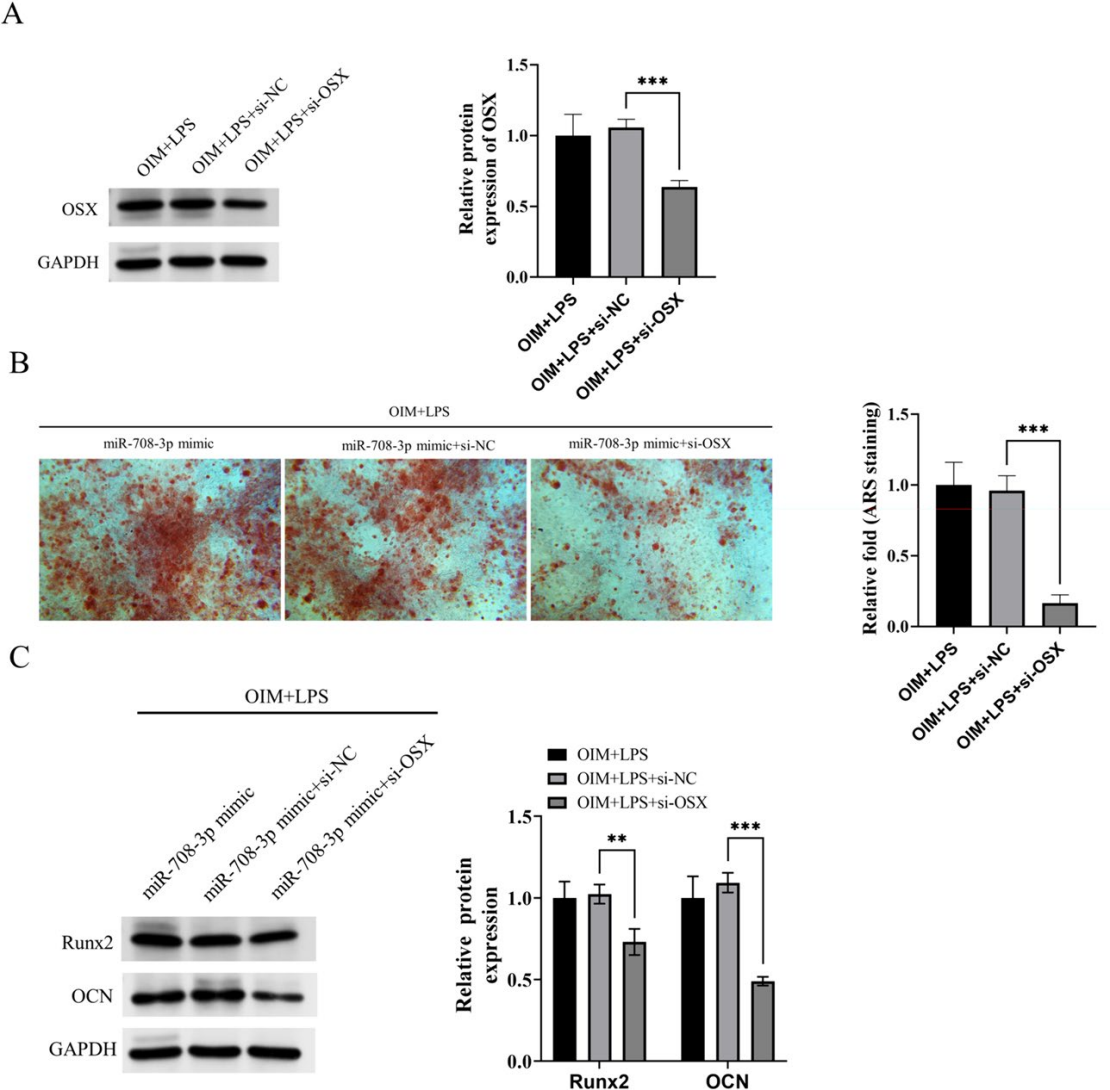
miR-708-3p promoted osteoblast differentiation of hPDLSCs by increasing OSX transcription in microenvironment of PD. **A** The protein levels of OSX in hPDLSCs in each group. **B** The mineralization levels of hPDLSCs in each group. **C** The protein levels of Runx2 and OCN in hPDLSCs in each group. The samples were from the same experiment, and the gel/imprint was processed in parallel. All stripes were cropped

To investigate the impact of OSX on hPDLSCs osteogenic differentiation, we downregulated its expression in hPDLSCs using si-OSX (Figs. 5A). The results showed that inhibition OSX notably reversed the promotive effects of miR-708-3p overexpression on hPDLSCs osteogenic differentiation in microenvironment of PD (Fig. 5B–5C). Taken together, miR-708-3p increased H3K4me2 methylation to enhance OSX transcription, and thus promoted hPDLSCs osteoblast differentiation in PD.

## Discussion

Periodontitis (PD) is a prevalent oral disease that destroys the tissues that support the teeth [25]. Conventional PD treatments focus on eliminating pathogens, controlling inflammation, and modulating immunity [26]. However, their efficacy remains unsatisfactory. The inflammation resulting from PD microenvironment can inhibit osteogenic differentiation of MSCs [27]. Although LSD1 has been found to regulate the inflammatory response in sepsis and breast cancer, little is known about its interaction with dental diseases [28]. Our findings revealed that LSD1 was downregulated in hPDLSCs in microenvironment of PD, and that its expression was negatively regulated by miR-708-3p through binding with it. Therefore, we aim to investigate the mechanism by which miR-708-3p targetedly regulates LSD1 to impact hPDLSCs osteogenic differentiation in PD.

Inflammatory conditions are a leading cause of immune disorders and PD [29]. In addition, aberrant production of proinflammatory cytokines can negatively affect the osteogenic differentiation of MSCs [30]. Previous studies have shown that LSD1 is recruited to injured tissues where it increases the expression of inflammatory cytokines and triggers inflammatory responses [31]. In the present study, we treated hPDLSCs with LPS to simulate an inflammatory microenvironment of PD. To investigate the impact of LSD1 on PD, we transfected hPDLSCs with si-LSD1 to reduce its expression. Subsequently, we applied ARS staining to evaluate osteogenic differentiation and observed an increased ARS staining in hPDLSCs transfected with si-LSD1. In addition, silencing LSD1 resulted in elevated expression of Runx2 and OCN. Runx2 plays a vital role in promoting the osteogenic differentiation and maturation of osteoblasts [32]. Overexpression of RUNX2 in osteoblasts in transgenic mice leads to increased osteogenic differentiation [33]. OCN is a key regulator of the mineralization process that takes place during the latter stages of osteogenic differentiation [34]. In summary, our findings suggest that inhibition of LSD1enhanced hPDLSCs osteogenic differentiation in PD.

Controlling the expression of LSD1, miRNAs have demonstrated the ability to regulate both the viability and differentiation of MSCs [35]. Furthermore, these small molecules have been demonstrated to be critical in detecting and assessing the severity of PD [36]. Using TargetScan databases, miR-708-3p was predicted as a potential miRNA candidate of LSD1. Notably, we found that miR-708-3p was predominantly expressed in the nucleus of hPDLSCs. Luciferase reporter assays confirmed that miR-708-3p directly targeted LSD1. Moreover, LPS treatment decreased miR-708-3p expression, whereas osteogenic induction increased it. In recent years, the biological functions of miR-708-3p have been studied. Lee et al*.* found that miR-708-3p could repress breast cancer by alleviating idiopathic pulmonary fibrosis [37]. However, its precise role in PD remains unknown. To investigate the effect of miR-708-3p in PD, we transfected hPDLSCs with miR-708-3p mimic. Subsequent analysis revealed that overexpression of miR-708-3p led to the promotion of osteogenic differentiation. Thus, miR-708-3p appears to encourage osteogenic differentiation by targeting LSD1 transcription.

The modification of histone methylases and demethylases through post-translational mechanisms has garnered growing interest in the field of PD research [38]. LSD1 has been demonstrated to demethylate H3K4me2, leading to increased expression of Runx2, ALP, and OSX, and ultimately promoting the potential for osteogenic differentiation of MSCs [39]. Our findings reveal that LSD1 modulates the transcription of OSX through demethylation of H3K4me2. As an osteoblast-specific transcription factor, OSX plays a critical role in promoting osteogenesis, formation, and reconstruction of teeth [40]. We next conducted Chip and western blot assay, and confirmed that miR-708-3p increased OSX transcription through LSD1-mediated H3K4me2 demethylation. To confirm the impact of miR-708-3 regulatory role on hPDLSCs osteogenic differentiation, we conducted a transfection of si-OSX in miR-708-3p-overexpressed hPDLSCs. Our findings revealed that the promotive effects of miR-708-3p on hPDLSCs osteogenic differentiation in the PD microenvironment were reversed upon silencing OSX.

In conclusion, our research demonstrated that miR-708-3p bound to LSD1, resulting in upregulated H3K4me2 methylation and the subsequent increasing OSX expression. This promoted osteogenic differentiation of hPDLSCs within a PD microenvironment. This study provides a pioneering perspective on the molecular mechanisms underpinning PD and offers an innovative therapeutic approach for the treatment of PD.

## Data Availability

The datasets used or analyzed during the current study are available from the corresponding author on reasonable request.
