# Correction: miR-708-3p targetedly regulates LSD1 to promote osteoblast differentiation of hPDLSCs in periodontitis

**DOI:** 10.1007/s10266-024-00983-5

**Published:** 2024-07-26

**Authors:** Qing Shao, ShiWei Liu, Chen Zou, YiLong Ai

**Affiliations:** 1 Department of Orthodontics, Foshan Stomatological Hospital, School of Stomatology and Medicine, Foshan University, No.5 Hebin Road, Chancheng District, Foshan, 528000 Guangdong China; 2 Department of Stomatology, Foshan First People’s Hospital, Foshan, 528000 Guangdong China

**Correction: Odontology** 10.1007/s10266-024-00963-9

The article “miR-708-3p targetedly regulates LSD1 to promote osteoblast differentiation of hPDLSCs in periodontitis”, written by Qing Shao, ShiWei Liu, Chen Zou and YiLong Ai, was originally published electronically on the publisher’s internet portal on 03 July 2024 without open access. With the author(s)’ decision to opt for Open Choice the copyright of the article changed on 9 July 2024 to © The Author(s) 2024 and the article is forthwith distributed under a Creative Commons Attribution 4.0 International License, which permits use, sharing, adaptation, distribution and reproduction in any medium or format, as long as you give appropriate credit to the original author(s) and the source, provide a link to the Creative Commons licence, and indicate if changes were made. The images or other third party material in this article are included in the article’s Creative Commons licence, unless indicated otherwise in a credit line to the material. If material is not included in the article’s Creative Commons licence and your intended use is not permitted by statutory regulation or exceeds the permitted use, you will need to obtain permission directly from the copyright holder. To view a copy of this licence, visit http://creativecommons.org/licenses/by/4.0.

